# Encephalitic Arboviruses of Africa: Emergence, Clinical Presentation and Neuropathogenesis

**DOI:** 10.3389/fimmu.2021.769942

**Published:** 2021-12-23

**Authors:** Robyn S. Klein

**Affiliations:** Center for Neuroimmunology & Neuroinfectious Diseases, Departments of Medicine, Neuroscience, and Pathology & Immunology, Washington University School of Medicine, St. Louis, MO, United States

**Keywords:** alphavirus biology, neuroimmunology, Flavivirus, Africa, CNS

## Abstract

Many mosquito-borne viruses (arboviruses) are endemic in Africa, contributing to systemic and neurological infections in various geographical locations on the continent. While most arboviral infections do not lead to neuroinvasive diseases of the central nervous system, neurologic diseases caused by arboviruses include flaccid paralysis, meningitis, encephalitis, myelitis, encephalomyelitis, neuritis, and post-infectious autoimmune or memory disorders. Here we review endemic members of the *Flaviviridae* and *Togaviridae* families that cause neurologic infections, their neuropathogenesis and host neuroimmunological responses in Africa. We also discuss the potential for neuroimmune responses to aide in the development of new diagnostics and therapeutics, and current knowledge gaps to be addressed by arbovirus research.

## Introduction

Recent studies indicate that climate changes in Africa may lead to a shift in vector-borne diseases from malaria to arboviruses due to differential effects of warming temperatures on the mosquito species that transmit these pathogens to humans [summarized in (1)]. Thus, neurotropic arboviruses that are transmitted by *Aedes aegypti*, and cycle between wildlife and livestock or humans in west sub-Saharan Africa, are likely to emerge in other areas of Africa where the current climates supports *Anopheles gambiae* transmission of malaria (2, 3). Recent epidemics of yellow fever (YFV) and Rift Valley fever (RVFV) viruses in Nigeria and Uganda (4, 5), respectively, and emergence of West Nile virus (WNV) in the Darfur region (6) are consistent with these predictions. In addition, the United Nations estimates suggest an increase in global population of 37% by 2050 (7), which facilitates transmission of vector-borne diseases through higher population densities and international travel. While the majority of infections with neurotropic arboviruses are asymptomatic, many persons develop flu-like symptoms that progress to neuroinvasive diseases in approximately half of symptomatic patients. In addition, 50-70% of survivors of CNS arboviral infection go on to develop neurocognitive and neuropsychiatric disorders that worsen over time (8). In this subsection, we will review the epidemiology, pathophysiology, and value of neuroimmune changes in diagnostics and therapeutics of medically important, African mosquito-borne neurotropic arboviruses. We will also provide current knowledge gaps and perspectives regarding future research in neurotropic arboviruses.

## Overview of African Mosquito-Borne Arboviruses That Induce Neuroinvasive Diseases in Humans

The etiologic agents of arboviral neuroinvasive diseases occur within three virologic genera: *Flaviviridae*, *Togaviridae*, and *Bunyaviridae*. The Phlebovirus RVFV (*Phenuiviridae* family) has been recently and extensively reviewed (9–14). Categorization of these RNA viruses, their key attributes, types of neuroinvasive diseases they cause, in addition to geographic epidemiology, and pathophysiology for medically relevant *Flaviviridae* and *Togaviridae* family members are summarized below (see **Table 1**).

**Table 1:** African arboviruses: vectors, geographical distribution, and the illnesses they cause in adults.

Family	Virus	Vector	Geographical distribution	Systemic illnesses	Neurological diseases
Flavivirida**e**	WNV	Mosquito (*Culex)*	Africa, Mediterranean region, Central Asia, India, Europe, North, Central and South Americas	Flu-like illness	Meningitis, flaccid paralysis, encephalitis, myelitis, memory disorders, Parkinsonism
	ZIKV	Mosquito *(Aedes)*, Sexual transmission	Africa, India, Southeast Asia, Carribean islands, Central, North and South Americas	Flu-like illness with arthralgias, conjunctivitis	Meningoencephalitis, ADEM, GBS, memory disorders
	DENV	Mosquito *(Aedes)*,	Africa, the Americas, the Eastern Mediterranean, South-East Asia and the Western Pacific	Fever, headache, pain behind the eyes, muscle pain, fatigue, nausea, vomiting, rash, bleeding hemorrhagic fever/shock	Encephalopathy, encephalitis, Guillain-Barre syndrome, transient muscle dysfunctions, neuro-ophthalmic involvement
Togaviridae	CHIKV	Mosquito *(Aedes)*	Subsaharan Africa	Fever, rash, arthralgias, myalgias	Rare encephalitis, GBS
	SINV	Mosquito (*Culex)*	Northeastern, Central and Southern Africa	Fever, rash, arthralgias, myalgias	Rare encephalitis

WNV, West Nile virus; ZIKV, Zika virus; DENV, dengue virus; CHIKV, Chikungunya virus; SINV, Sindbis virus.

### Flaviviridae

**Figure 1:**
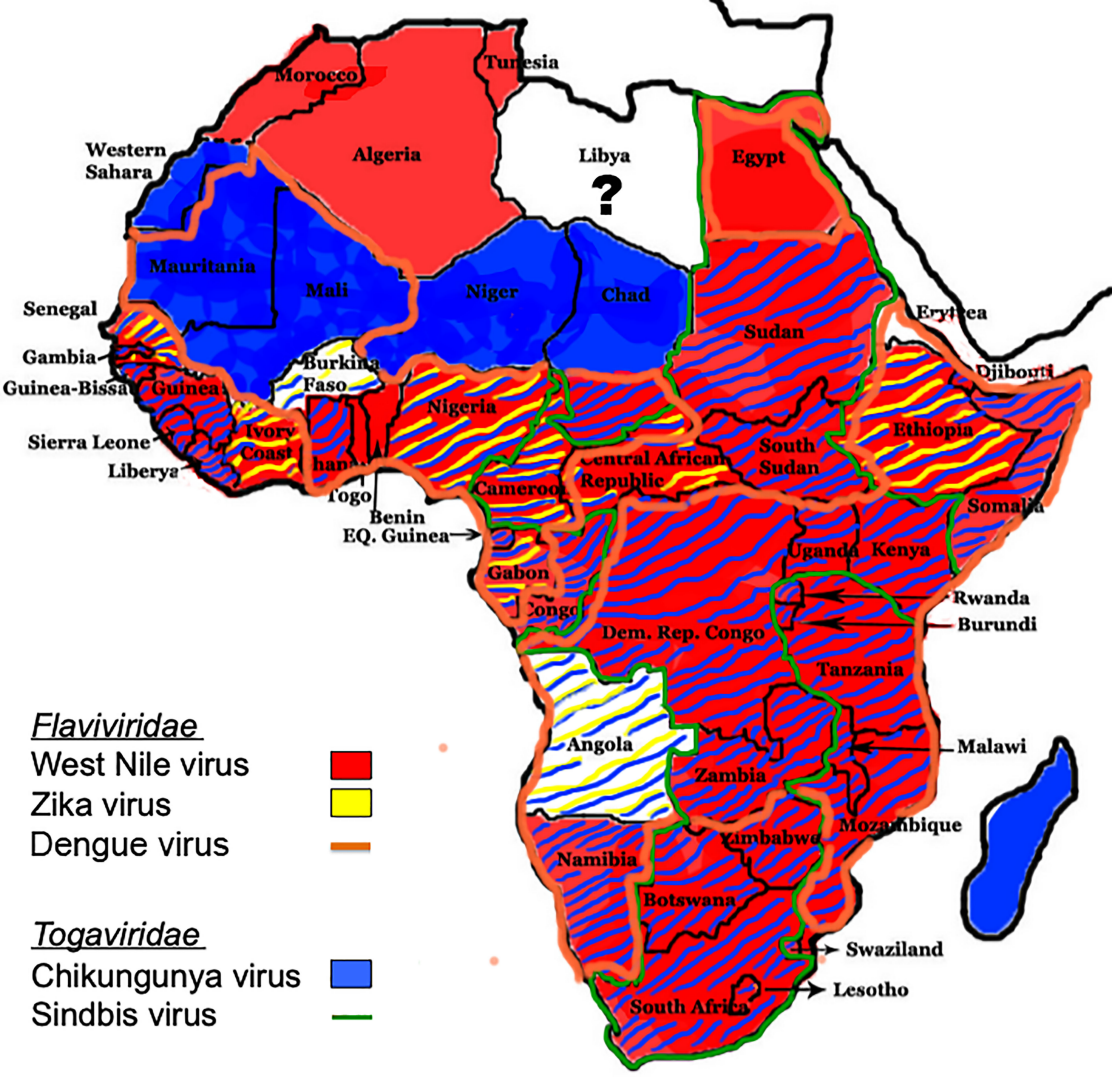
Distribution of flaviviruses and alphaviruses in Africa. The distribution of *Culex-* and *Aedes*-transmitted flaviviruses WNV, ZIKV, and DENV, and *Aedes*- and *Culex-*transmitted alphaviruses CHIKV and SINV, respectively, throughout Africa are shown (17).

Members of the *Flaviviridae* family of viruses are enveloped, with a positive single-strand RNA genome of 9-13 Kb with that replicates as a single open reading frame (ORF) with genes for three structural and seven nonstructural (NS) proteins (15). Structural proteins, which comprise the virion, consist of the viral capsid and the envelope glycoproteins. NS proteins are essential for replication of the viral genome, transcription and translation of viral genes, viral assembly, and may modulate immune function to promote infection and dissemination within humans. Phylogenetic trees indicate that all vector-borne flavirviruses originated in Africa, likely from non-vectored mammalian viruses (16). Medically important, neurotropic flaviviruses that cause CNS disease in Africa are transmitted by *Culex* (West Nile encephalitis viruses; WNV), and *Aedes* (Zika virus; ZIKV, Dengue virus; DENV) mosquito species (17). WNV was first isolated from a febrile patient in the West Nile district of Uganda in 1937, while ZIKV was first identified in a rhesus monkey from the African regions in Kampala, Uganda, in the Zika forest in 1947 (18, 19). A DENV epidemic was first reported in 1823 in the Zanzibar Islands (20). WNV human cases occur in most African countries throughout the continent with the exception of the western Sahara desert, Mauritania, Mali, Burkina Faso, Niger, northern Chad, Libya, and Angola (17) (**Figure 1**). ZIKV outbreaks in humans have occurred in only nine countries: Senegal, Cote D’Ivoire, Burkina Faso, Nigeria, Cameroon, Gabon, Central African Republic, Ethiopia, and Angola (**Figure 1**). DENV, which exists as four closely related but distinct serotypes, is endemic in almost all African countries, with the exception of Morocco, Algeria, Tunesia, Western Sahara, Niger, Chad, Sudan, Gambia, Guinea-Bissa, Guinea, Sierra Leone, Liberya, Ivory Coast, Central African Republic, South Sudan, Congo, Burundi, Botswana, Zimbabwe, Swaziland, and Lesotho (**Figure 1**). Both WNV and ZIKV may also be transmitted *via* transfusion of human blood products, and ZIKV can also be transmitted *via* sexual contact, primarily with males.

WNV and ZIKV are neurotropic viruses that can cause acute flu-like illnesses with fever, headache, rash, pharyngitis, diarrhea, arthralgias, conjunctivitis, and myalgias (21). Most humans infected with WNV or ZIKV are asymptomatic, however 20-25% of cases become symptomatic, and in those infected with WNV, approximately half of these patients will develop neuroinvasive diseases including meningitis, encephalitis, myelitis, and flaccid paralysis. Vertical transmission leading to teratogenic effects of ZIKV during pregnancy is also well documented with approximately 20% of affected fetuses exhibiting morphological abnormalities by ultrasound (e.g., microcephaly or brain calcifications), whereas the vast majority exhibit no overt clinical manifestations at birth (22–24) Diagnostic tests include assessment of serum or CSF virus-specific IgM or PCR detection of viral RNA (21). Reported neuroinvasive diseases in the setting of ZIKV infection include cases of meningitis, encephalitis, and encephalomyelitis. Patients with a concurrent or past history of ZIKV systemic infection may also present with Guillain-Barré syndrome (GBS) and myeloradiculitis, which may respond to intravenous IVIG (25, 26). Neurologic and functional disability associated with these flaviviruses can also continue to cause morbidity in patients after recovery from acute illness. Studies of WNV survivors report that in 50-70% of survivors exhibit symptoms that persist and worsen over time including confusion, muscle weakness, concentration difficulties, parkinsonism, and memory impairments, especially in the realm of visuospatial memory (27). Severe cases of ZIKV-induced systemic disease may also lead to neurocognitive deficits, daily headaches, and chronic inflammatory demyelinating polyneuropathies that may persist for years (28–32).

Neurological diseases associated with DENV infection were first reported in 1976 as atypical symptoms of dengue infection, and their incidence rates have varied from 0.5% to 20% (33). Neurological symptoms associated with DENV infection have increasingly been reported in both children and adults, and include encephalopathy due to hepatic failure or metabolic disorders, encephalitis due to direct viral invasion, Guillain-Barre syndrome or transient muscle dysfunctions, and neuro-ophthalmic involvement (34).

Dengue serotypes 2 and 3 are most commonly associated with neurological symptoms (35, 36). Although DENV is not primarily neurotropic, a recent study utilizing genome analysis and characterization of DENV type 2 (DENV-2) strains isolated from cerebrospinal fluid (CSF) and/or serum of patients with dengue encephalitis revealed that the DENV-2 isolates belonged to a new clade of cosmopolitan genotype that are genetically close to strains identified in China, South Korea, Singapore, Malaysia, Thailand, and the Philippines (37). As DENV does not invade the CNS when inoculated peripherally in mice, few studies have determined its route of neuroinvasion or CNS immune responses that exert virologic control.

**Figure 2:**
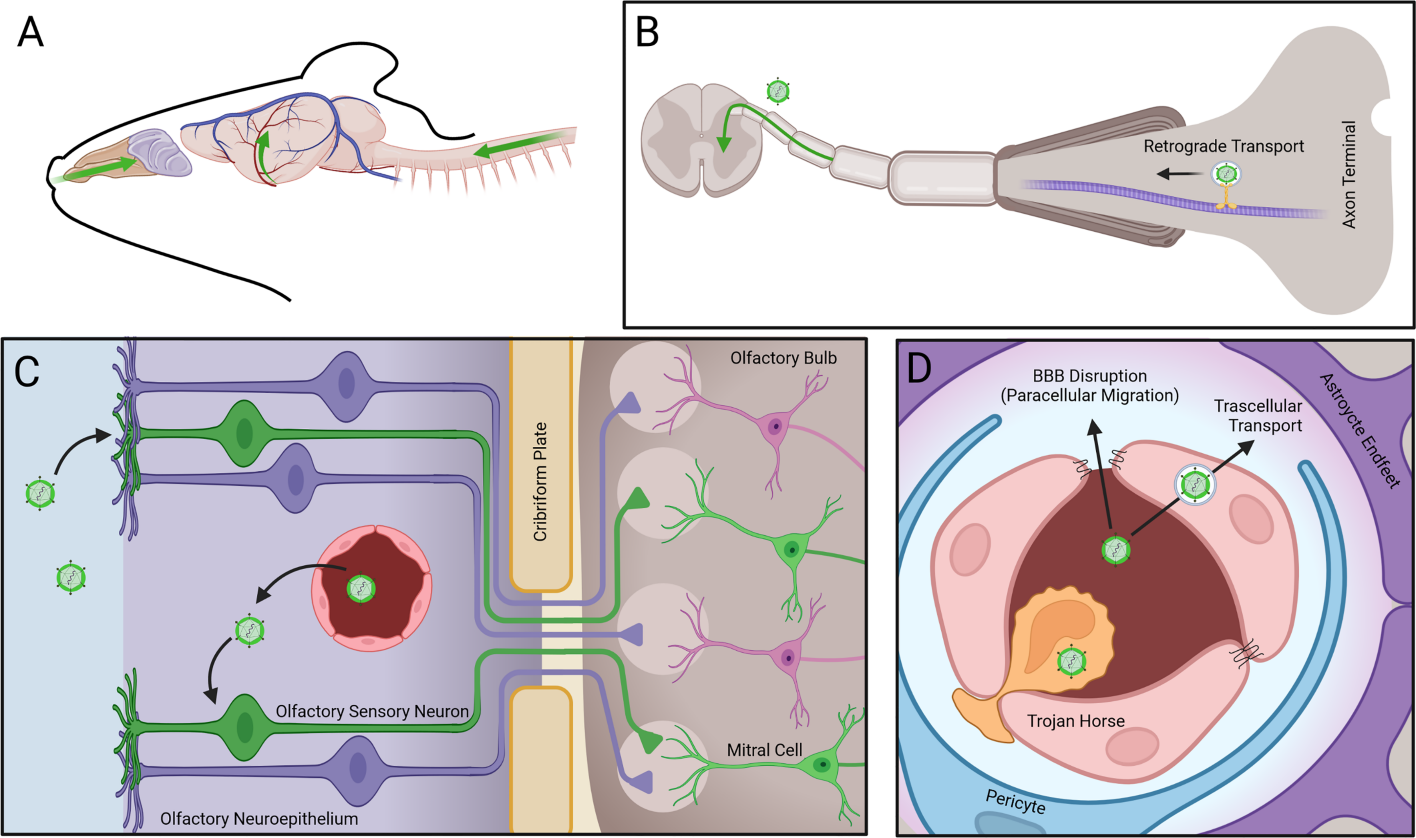
Mechanisms of arbovirus entry into the CNS. Arboviruses enter the CNS via three routes: **(A, B)** Retrograde transport of virus along axon microtubules (MT) of peripheral neurons allows entry into the spinal cord. **(C)** Infection of olfactory sensory neurons (OSNs) in the olfactory neuroepithelium (ONE) following infection from fenestrated capillaries (FC) allows viral intra-axonal migration through the cribiform plate (CP), followed by transynaptic infection of mitral cells (MC) at the glomeruli (G) of the olfactory bulb (OB). **(D)** Virus entry through the blood brain barrier (BBB) occurs via transcellular transport of virions, paracellular migration of virions following disruption of tight junctions (TJs), or via transmigration of infected leukocytes.

The pathogenesis of WNV and ZIKV CNS infections in humans is incompletely defined, although excellent mouse models have illuminated mechanisms of immune control in the periphery and central nervous system (CNS) (38), routes of viral neuroinvasion (39–45), features of virus-induced encephalitis (46, 47), and processes that induce post-infectious neurocognitive sequelae (48–52). Neuroinvasion can occur hematogenously as free virions or within CNS infiltrating immune cells, and *via* retrograde transport along sensory axons from sites of mosquito inoculation in the periphery (53) (**Figure 2**). The brain vasculature exhibits specializations that prevent paracellular and transcellular entry of cells, pathogens, and metabolites. These occur at the post-venular and capillary levels and include tight and adherens junctions (TJ and AJ), low levels of leukocyte adhesion molecules, and low rates of transcellular vesicle trafficking (transcytosis). Rho GTPase signaling pathways that control the assembly and disassembly of endothelial cytoskeletal proteins regulate TJ integrity, which affects BBB permeability. During acute infection with flaviviruses, local expression of BBB destabilizing cytokines activate the RhoA/ROCK/pMLC signaling pathway, which induces stress fiber formation that disrupts TJ and increases paracellular permeability. Increased blood-brain barrier (BBB) permeability during acute infection has also been linked to rising levels of NS1 within the blood, which correlate with severity of disease. NS1 is secreted from virally infected cells and may up-regulate the expression of cathepsin L and endoglycosidase heparanase in brain endothelial cells, leading to the degradation of glycocalyx-like layer (EGL) components with consequent damage to BBB integrity (54, 55). Flavivirus traversal across the BBB is believed to occur *via* paracellular and transcellular routes, the latter of which includes delivery by leukocytes (56). Neuroimaging during the acute setting may be normal or reveal BBB disruption, which is associated with more severe outcome (57).

Once WNV or ZIKV enter the CNS, they infect and injure neurons (or neuroprogenitor cells in the case of ZIKV) through direct (virus infection-induced) and indirect (immune-mediated) mechanisms (58, 59). Microscopic examination of the post-mortem CNS specimens may reveal neuronal cell death, microglial activation, infiltrating macrophages, and accumulation of CD4^+^ and CD8^+^ T cells (60, 61). Depending on the flavivirus, these lesions can occur in the brainstem, cerebral cortex, hippocampus, thalamus, cerebellum or spinal cord. While it is well established that both humoral and cell-mediated immune responses critically control viral replication in peripheral tissues, virologic control within the CNS predominantly requires the infiltration of antiviral mononuclear cells (62–64). Viral replication within neurons is detected by the cytoplasmic RNA helicases RIG-I and MDA5, which signal through the adaptor protein mitochondrial antiviral signaling protein (MAVS) to promote antiviral gene expression and proinflammatory proteins, including T cell chemoattractants in both neurons and activated astrocytes and microglia (38). Antiviral, CD8 T cells recruited to the acutely infected CNS can eliminate virus from neurons *via* non-cytolytic effects of interferon(IFN)γ (65). Subpopulations of effector CD8 T cells persist as resident memory T cells (Trm) that continue to express IFNγ, which maintains microglia activation (49). During acute infection, infected neurons and activated microglia exhibit upregulation of complement proteins (52), which have been implicated in the maintenance or disruption of neural networks (66). Studies in WNV- and ZIKV-infected mice show complement- and microglia-mediated elimination of synapses within the trisynaptic circuit of the hippocampus (52) is associated with defects in spatial and other forms of learning and memory. Studies in humans that succumbed to WNV show similar loss of synapses. Macrophage delivery of interleukin(IL)-1 has been shown to maintain a proinflammatory state *via* direct effects on neural stem cells within the neurogenic niche of the hippocampus, promoting decreased neurogenesis in favor of production of neurotoxic, reactive astrocytes that prevent synapse repair, and persist long-term (67). Future studies are needed to determine whether these processes occur and may be targeted in humans to prevent or treat neurocognitive sequelae after recovery from neurotropic flavivirus infection.

### Togaviridae

Members of the *Togaviridae* family of viruses are small, enveloped viruses with single-stranded positive-​sense RNA genomes of 10–12 kb that encode five structural and four NS proteins (68). Two thirds of the genome of alphaviruses encodes the non-structural polyprotein(s) in a single ORF immediately after a 5′-non-coding region. Overlapping with the 3′-end of the non-structural ORF, there is a promoter for transcription of a subgenomic mRNA from which the structural polyprotein is translated (69). The genus Alphavirus comprises a large group of medically important mosquito-borne viruses that are transmitted by *Aedes* (Chikungunya virus; CHIKV), and *Culex* (Sindbis virus; SINV) (17). Phylogenetic tree analyses suggest that alphaviruses likely originated from an aquatic habitat, from ancestral strains such as the Southern elephant seal virus and other fish viruses, followed by spread to New and Old World (70). The first reported CHIKV and SINV outbreaks occurred in Tanzania and Egypt, respectively, in 1952 (71). Seroprevalence for CHIKV is found throughout sub-Saharan Africa (**Figure 1**), while SINV occurs in a geographical area that spans from South Africa to Egypt and from Cameroon to Kenya (16) (**Figure 1**).

CHIKV and SINV generally cause febrile syndromes with rashes and joint pain, and are only occasionally associated with neurologic diseases. CHIV infection is asymptomatic in up to 25% of human infections, with symptomatic cases presenting with fever, headache, myalgia, arthritis, conjunctivitis, nausea/vomiting, maculopapular rash and incapacitating bilateral and symmetric polyarthralgia, which may relapse or persist for months to years (72). Rare neurologic complications include seizures, acute flaccid paralysis, Guillain-Barré syndrome, cranial nerve palsies, myelitis, encephalopathy, and meningoencephalitis (73). Persons at risk for CNS disease include neonates exposed intrapartum, older adults (e.g., > 65 years), and persons undergoing immunosuppression for solid organ transplant (74). Case fatality rate for CHIKV encephalitis ranges from 4-28%, with higher rates mostly in older adults. Electroencephalogram in patients with neurologic signs may exhibit slow background activity and generalized epileptiform discharges, while brain MRI may show bilateral white matter hyperintensities and/or focal encephalitis. Postmortem brain examination of a patient who succumbed to CHIKV encephalitis revealed lymphocytic infiltrates with focal necrosis in the hippocampus, frontal lobes and medulla oblongata (75). While many SINV infections are asymptomatic, cases usually present with a maculopapular, pruritic exanthema over the trunk and limbs, mild fever, and arthralgia, particularly in wrists, hips, knees, and ankles, sometimes accompanied by nausea, general malaise, headache, and myalgia (76). Patients can experience persistent joint manifestations that continue for months or years, and in rare cases as a chronic arthritis. SINV is known to cause neurologic disease in horses (77), but human cases are extremely rare.

The mechanisms of CHIKV and SINV neuroinvasion in humans are unknown; however, animal models suggest entry may occur *via* invasion of brain endothelial cells and retrograde axonal transport, respectively (78) (**Figure 2**). Studies examining CHIKV and SINV infection of the CNS in murine models report multiple sites of neuronal and astrocyte infection progressing to cell death *via* caspase-mediated pathways, with microgliosis and perivascular cuffs (75, 79–82). Similar to reports in human cases of CHIKV encephalitis (75), neuronal degeneration in the hippocampus and lymphocytic meningitis is also observed in animals. As with flavivirus encephalitis, CHIKV RNA is detected in the brain by pattern recognition receptors, such as toll-like receptor(TLR)-3, that upregulate innate immune antiviral molecules that can reduce viral replication (83). While increased expression of the T cell cytokine IFNγ has been observed in animal models, mechanisms of T cell trafficking and virologic control within the brain have not been investigated. Likewise, there have been no reports of long-term follow-up in survivors of CHIKV neurologic diseases.

## Can Neuroimmune Responses Aide in Diagnostics and/or Therapeutics?

The diagnosis of arboviral neuroinvasive diseases requires virus-specific assays so that novel therapies, such as antibody-based therapeutics, and patient prognoses can be accurately administered. Studies attempting to identify virus-specific innate or adaptive immune pathways *via* genomic approaches in animal models have been instrumental in identifying the critical antiviral pathways that control and clear virus (84, 85), but have failed to support the use of pathway analysis for diagnostic purposes. Knowledge regarding the status of BBB permeability may also be critical for treating acute neuroinvasive diseases. For example, animal studies examining patterns of BBB function throughout the course of flavivirus encephalitis indicate that induction of interferon responses may promote BBB closure *via* Rac1-mediated effects on TJ integrity (40, 45). Thus, use of anti-viral antibodies for CNS infection may have a limited window of penetration. While there are currently no treatments that limit the replication of specific arboviruses in the CNS, anti-inflammatory treatments, including corticosteroids, have been used in patients with chorioretintis, encephalitis or myelitis (86–88). New anti-inflammatory compounds are also under development (89).

## Knowledge Gaps for Future Research

One of the challenges for limiting arboviral neuroinvasion and dissemination within the CNS is the incomplete knowledge regarding virus-specific entry receptors expressed at the BBB and by neural cells, including those involved in trans-synaptic spread between CNS regions. Entry receptors postulated to be involved in flavivirus entry include α_v_β_3_ integrins, C-type lectin receptors (CLR), phosphatidylserine receptors TIM (T-cell immunoglobulin and mucin domain) and TYRO3, AXL and MER (TAM) family of receptor tyrosine kinases (90, 91). Attachment and entry receptors for CHIKV include glycosaminoglycans (GAGs), T-cell immunoglobulin and mucin 1 (TIM-1), and the cell adhesion molecule Mxra8 (92). While many of these receptors are expressed at CNS barriers and within the parenchyma, the demonstration these receptors are required for brain endothelial and neural cell entry is currently lacking. There is also a dire need to identify biomarkers that identify survivors of arboviral neuroinvasive diseases at risk for neurological sequelae, including neurocognitive impairments. Post-infectious neurocognitive sequelae modeled in murine models show benefit from administration of anakinra, a USFDA approved medication that targets the IL-1R for the treatment of rheumatoid arthritis, during acute encephalitis (93). Given the essential role of the IL-1R, in CNS virologic control, it is unclear whether the risk-benefit ratio supports use of this drug in humans with arboviral encephalitis. Future studies are needed to better identify and define safe therapeutic targets to limit the entry and dissemination of neurotropic arboviruses, and to prevent the development of neuroimmune processes that contribute long-term sequelae.

## Author Contributions

The author confirms being the sole contributor of this work and has approved it for publication.

## Funding

This work was supported by NIH grants R35NS122310.

## Conflict of Interest

The author declares that the research was conducted in the absence of any commercial or financial relationships that could be construed as a potential conflict of interest.

## Publisher’s Note

All claims expressed in this article are solely those of the authors and do not necessarily represent those of their affiliated organizations, or those of the publisher, the editors and the reviewers. Any product that may be evaluated in this article, or claim that may be made by its manufacturer, is not guaranteed or endorsed by the publisher.
